# Antimicrobial activity, mechanical and thermal properties of cassava starch films incorporated with beeswax and propolis

**DOI:** 10.1007/s13197-023-05878-x

**Published:** 2023-11-16

**Authors:** María Carolina Betancur-D´Ambrosio, Carmen Elena Pérez-Cervera, Cindy Barrera-Martinez, Ricardo Andrade-Pizarro

**Affiliations:** 1 Agro-Industrial Engineering Program, Pontificia Bolivariana University, Montería, Colombia; 2 Research and Advanced Engineering, Ford Motor Company, Dearborn, USA; 3 Department Food Engineering, Universidad de Córdoba, Montería, Colombia

**Keywords:** Edible films, Biopolymers, Mechanical properties

## Abstract

Edible films can be formed from different polymeric compounds. The use of starch has gained extra value; because it can be used in combination with plasticizers and lipids, helping to improve mechanical properties. Besides, with the addition of an antimicrobial, the function of these films can be extended. The objective of this research was to evaluate the effect of native cassava starch, beeswax and ethanolic propolis extract (EPE) on the mechanical, thermal and inhibitory properties against the *Aspergillus niger* fungus. An experimental Box-Behnken design with three factors: cassava starch concentration (2–4%*w/v*), beeswax (0.5–0.9%*w/w*) and EPE (1–4%*v/w*) was used. The films obtained were opaque and with low mechanical properties. EPE concentration affected tensile strength, elongation at break (EB) and Young’s modulus (YM), and cassava starch content only affected EB and YM. In thermal properties, the weight loss was affected by the cassava starch-beeswax interaction, where the most loss occurred at high levels of these factors in the temperature range of 200–360 °C. The films reduced the growth of the *Aspergillus niger* by 51%, where the beeswax-EPE interaction had a significant positive effect. The characteristics of the developed films suggest that they would be more acceptable as fruit and vegetable coatings.

## Introduction

From the moment of harvest, fruit and vegetable products undergo a series of physicochemical changes, due to internal processes that condition storage and type of packaging, which can negatively affect their organoleptic, nutritional and microbiological properties. These changes during postharvest are especially due to interactions between the food and its environment, causing the migration of different components within the food, leading to the loss of moisture and/or some gases. In addition, horticultural products are easily attacked by the proliferation of pathogenic microorganisms, especially fungi, such as *Aspergillus niger, Rhizopus orizae, Botrytis cinerea*, among others, affecting the shelf life of the product and thus causing large postharvest losses (Lin and Zhao 2007; Pandey et al. 2022).

Different technologies have been developed to mitigate postharvest losses of fruit and vegetable products, among which is the application of edible films and coatings, which is an alternative that is in line with market demands in terms of food preservation. Edible coatings can replace and/or strengthen the natural layers of fruits and vegetables to prevent moisture losses and to control exchange gases involved in respiration processes, such as oxygen, carbon dioxide and ethylene. Moreover, edible films and coatings may also be used as a vehicle to incorporate various ingredients (Andrade et al. 2012; Nayak et al. 2019)**.**

Edible films and coatings are mainly elaborated with polymers (polysaccharides, proteins, and lipids), solvents and plasticizer. Starch is a polymer of natural origin, consisting of amylose and amylopectin, which can be obtained from several renewable sources, such as cassava, potato or corn. Starch films are edible, odorless, tasteless, colorless and semi-permeable to gases, moisture, lipids and flavor components, which are essential for effective food packaging materials. Cassava starch contains approximately 17 % amylose, which facilitates its film formation compared to other vegetable starches. Moreover, it is appreciated for its paste transparency, low gelatinization temperature, and good gel stability (Mali et al. 2006; Ordoñez et al. 2021; Podshivalov et al. 2017).

While an edible film or coating can serve to control gas exchange between a food and its environment, the application of antimicrobial substances additionally provides protection against the proliferation of pathogenic microorganisms. Studies have shown that the use of substances such as propolis, oregano oil, rosemary oil, potassium sorbate, among others, have resulted in the inhibition of microbial growth, thus extending the advantages in the use of edible films and coatings (Ardjoum et al. 2021; Ochoa et al. 2017).

To evaluate the efficacy and quality of edible coatings, several parameters can be determined in fruits and vegetables covered during storage, or direct measurement of the films, including mechanical and thermal properties. An edible film must have mechanical properties that can maintain the integrity of the coating during handling, packaging, and transportation; in addition, to be resistant to rupture and abrasion, to reinforce the structure of the food and facilitate its handling and/or be flexible, with sufficient plasticity to adapt to possible deformations of the product without breaking (Andrade et al. 2012; Lin and Zhao 2007).

Propolis has demonstrated interesting biological properties such as antibacterial, antifungal, anti-inflammatory, and antioxidant (Bertotto et al. 2022; Bodini et al. 2013). Biopolymer-based films incorporated with propolis extract include matrices such as starch (Eskandarinia et al. 2018; Pérez-Vergara et al. 2020; Villalobos et al. 2017), pectin (Marangoni Júnior et al. 2022), gelatin (Bodini et al. 2013; Reyes et al. 2021), and chitosan (De Carli et al. 2022; Siripatrawan and Vitchayakitti 2016). Beeswax is the most commercially used natural wax with a wide range of applications in cosmetic products and food processing (Diyana et al. 2021), mainly due to its hydrophobic properties. Beeswax has been used in edible films and coatings made from gums (Haq et al. 2016; Saurabh et al. 2016), starch (Auras et al. 2009; Pérez-Vergara et al. 2020), chitosan (Hromiš et al. 2015), and gelatin (Zhang et al. 2018). However, there are quite limited studies reporting the characterization of starch-based films incorporated with beeswax and propolis. Therefore, the present study aims to investigate the effect of native cassava starch, beeswax, and ethanolic propolis extract on the mechanical and thermal properties and antimicrobial activity of cassava starch films.

## Materials and methods

### Raw materials

The native cassava starch was provided by “Almidones de Sucre S.A.S.”, the propolis was purchased from apiaries located in the municipality of Arboletes (Antioquia, Colombia). The strain of *Aspergillus niger* was supplied by the Microbiology Laboratory of the University of Córdoba (Montería, Colombia). For culture media, Potato Dextrose Broth and Potato Dextrose Agar were used, all provided by Scharlab (Barcelona, Spain).

### Extraction of ethanolic propolis extract (EPE)

EPE was obtained according to Bodini et al. (2013), with some modifications. 30 g of propolis were mixed with 100 mL of ethanol (80% *v/v*) and then stirred (500 r.p.m.) at 50 °C for 30 min. After extraction, the mixture was stored at 10 °C for 24 h and subsequently filtered through a Whatman grade 1 (I.C.T, S.L, Spain) filter paper. The filtered solution was employed as EPE.

### Film edible preparations

Film-forming solutions were prepared through gelatinization of cassava starch (2–4% *w/v*) at a temperature of 75 °C, with continuous stirring; the solutions were plasticized with glycerol (1.2% *w/w* of solution). Beeswax was first melted on a hot plate at 85 °C, and the molten wax was added to the solution at concentration of 0.5–0.9%. Then Tween 80 (20%) was added. Subsequently, this film-forming solution was cooled to room temperature and the EPE was added, applying continuous stirring. The solution was placed in an ultrasonic bath (1510R-DTH, Branson ultrasonic, Mexico) for 10 min, in order to eliminate air bubbles. The films were prepared using a solvent casting method. 115 g of the film-forming solution were poured into a polytetrafluoroethylene (Teflon®) molds (18.7 × 18.7 cm), and dried in a forced air oven (UN 55 plus, Memmert, Germany), at 40 °C for 48 h. The produced films were conditioned at 25 °C and 40% of relative humidity (RH) before further analysis.

### Edible films characterization

#### Film thickness

Sample thickness was measured using a digital micrometer (Tesa Technology, Renen, Switzerland) at ten random positions, and the average value was taken for the analysis of subsequent experiments.

#### Mechanical properties

The mechanical properties of the films were determined in a universal testing machine (Instron 3366; Instron Engineering Corp., Norwood, MA, USA), according to standard method ASTM D882, with some modifications. Film samples of 12.54 × 136 mm were prepared in a rectangular shape. The test condition of grip separation and cross-head speed was 50 mm and 12.5 mm/min, respectively. Tensile strength (TS, MPa), elongation at break (EAB, %), and Young’s Modulus (YM, MPa) were assessed. YM was the specific value of normal stress and normal strain. TS and EAB were calculated using Eqs. 1 and 2, respectively.

1 $$TS= \frac{{L}_{p}}{A}$$

where *L*_*P*_ is the film maximum force (N) that can withstand before breaking, and *A* is the film cross-sectional area (m^2^).

2 $$ EAB, \% = \frac{\vartriangle L}{L} \times 100 $$

where *ΔL* is the film extension length (mm) prior to breaking, and *L* is the film initial length (mm).

#### Thermal properties

The thermal properties of the films were assessed in a thermogravimetric analyzer (Mettler Toledo, TGA/DSC1, Schwerzenbach, Switzerland) which allows the recording of the weight loss as a function of temperature. The film samples were heated from 25 to 600 °C (heating rate: 10 °C/min), under a nitrogen gas flow rate of 30 mL/min.

#### Anti-fungal activity

The anti-fungal activity (*Aspergillus niger*) of starch film loaded with beeswax and propolis was evaluated by disc diffusion method reported by Pastor et al. (2010) with some modifications.

*Culture preparation*:* Aspergillus niger* was cultivated on potato dextrose agar (PDA) for 5 days at 25 °C. The spores were harvested using 5 mL of water. Finally, the spore suspension was adjusted to a final spore concentration of 10^5^ CFU/mL.

*Agar diffusion test*: The antifungal activity of prepared films was also carried out using the disc diffusion method with minor modification (Chollakup et al. 2020).

Antimicrobial activity was evaluated by contacting film disks of 20 mm in diameter with 10^5^ CFU/mL of tested fungi in PDA plates. Each plate was sealed with Parafilm® and incubated for 5 days at 20 °C. At the end of the incubation period, antimicrobial activity of the films was determined by counting spores using the *Neubauer improved* method. For the control sample, media inoculated in contact with film discs without propolis and wax were used. The percent inhibition of spore production was computed by the Eq. 3.

3 $$I, \%= \frac{{N}_{c}-{N}_{s}}{{N}_{c}} x 100$$

where N_c_ is the number of spores in control sample, N_s_ is the number spores in treated sample.

### Experimental design and statistical analysis

A three-factor Box-Behnken response surface design with three replicates at the center point was employed in this study, requiring a total of 15 experiments. The independent variables and their levels were: native cassava starch (2 and 4% *w/v*), beeswax (0.5 and 0.9% *w/w*) and ethanolic propolis extract (1 and 4% *v/w*). The response variables were: tensile strength, elongation at break, Young’s modulus, thermal properties and antimicrobial activity. Analysis of variance at the 5% significance level was used to identify statistically significant differences between the mean results associated with each property and treatment. Multiple range tests, using the Tukey test, were used to determine which mean results were significantly different. Statistical analysis was performed using the JMP 9.0.1 software (SAS Institute).

## Results and discussion

Native cassava starch films incorporated with beeswax and ethanolic propolis extract were found to be flexible, translucent, and easily removed from the casting plate.

### Mechanical properties

The Mechanical properties (Tensile strength (TS), elongation at break (EAB), and Young’s modulus (YM)) of native cassava starch films incorporated with beeswax and ethanolic propolis extract (EPE) are presented in Table 1.

**Table 1:** Mechanical properties of native cassava starch films incorporated with beeswax and ethanolic propolis extract

Run	Cassava starch, % *w/v*	Beeswax, % *w/w*	EPE, % *v/w*	TS, MPa	EAB, %	YM, MPa
1	3	0.9	4.0	4.51 ± 0.32	0.35 ± 0.01	12.88 ± 0.01
2	2	0.5	2.5	4.10 ± 0.62	0.31 ± 0.06	13.22 ± 0.10
3	2	0.7	1.0	5.93 ± 0.69	0.43 ± 0.03	13.79 ± 1.06
4	2	0.7	4.0	3.45 ± 0.57	0.41 ± 0.03	8.41 ± 0.03
5	4	0.9	2.5	2.13 ± 0.33	0.27 ± 0.01	7.88 ± 0.06
6	3	0.5	4.0	4.17 ± 0.49	0.44 ± 0.09	9.48 ± 0.02
7	4	0.5	2.5	3.45 ± 0.86	0.44 ± 0.05	7.84 ± 0.40
8	3	0.5	1.0	3.69 ± 0.10	0.35 ± 0.06	10.54 ± 0.10
9	2	0.9	2.5	1.87 ± 0.15	0.19 ± 0.04	9.84 ± 0.10
10	3	0.9	1.0	3.38 ± 0.67	0.15 ± 0.06	22.53 ± 0.06
11	4	0.7	1.0	4.66 ± 0.16	0.69 ± 0.04	6.75 ± 0.04
12	4	0.7	4.0	3.63 ± 0.12	1.06 ± 0.20	3.42 ± 0.04
13*	3	0.7	2.5	2.29 ± 0.79	0.21 ± 0.06	10.90 ± 0.06
14*	3	0.7	2.5	2.15 ± 0.01	0.18 ± 0.02	11.94 ± 0.14
15*	3	0.7	2.5	2.38 ± 1.16	0.31 ± 0.00	7.68 ± 0.02

^*^Center points

#### Tensile strength

Tensile strength values were between 1.87 and 5.93 MPa; similar results were reported for films made from cassava starch (Chollakup et al. 2020), sweet potato starch (Ballesteros-Mártinez et al. 2020), sugar palm (*Arenga pinnata*) starch (Sanyang et al. 2015), and native jicama starch (Wigati et al. 2022).

**Fig. 1:**
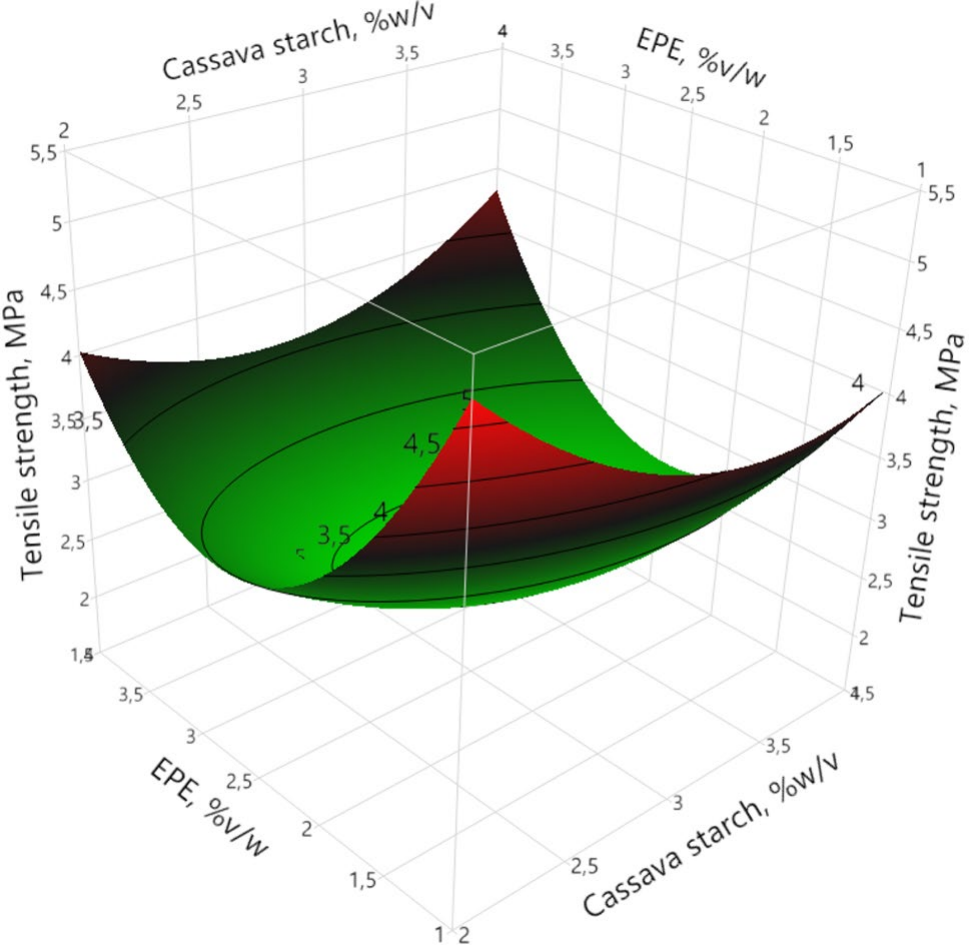
Response surface of tensile strength as a function of native cassava starch and ethanolic propolis extract (EPE)

Analysis of variance (ANOVA) shows that the tensile strength (TS) is influenced significantly (95%) by the quadratic factor of EPE (*p* < 0.0001), this means that the TS values decrease to a minimum point (1.59 MPa), and subsequently there was an increase in the TS (Fig. 1). As EPE concentration increases from 1.0 to 2.47%*v/w*, a decrease in TS was observed, which agrees with what was reported by Ardjoum et al. (2021), and Marques et al*.* (2021). The increase in hydrophobic compounds (flavonoids, phenolic acids and their esters especially) could lead to a reduction in the intermolecular interactions between starch chains, causing a reduction in the TS of cassava starch films (Marangoni Júnior et al. 2022). An increase in TS was observed as EPE concentration increased from 2.47 to 4.0%v/w, which is consistent with the findings of previous studies on films made from different matrices that incorporated propolis extract, including films made from chitosan (Siripatrawan and Vitchayakitti 2016), and PVA/starch (Mustafa et al. 2020). This could be due to the interactions of EPE compounds (resins, flavonoids, phenolic acids, and their esters) with the starch cassava. These components have polar characteristics and can interact with the hydrophilic groups of the starch molecules, which produces a stronger interfacial adhesion between the starch and the EPE which leads to a more efficient resistance to mechanical stress (Pastor et al. 2010; Siripatrawan and Vitchayakitti 2016).

#### Elongation at break

Elongation at break (EAB) values were between 0.15 and 1.06%, similar results were reported for films made from low-density polyethylene (LDPE)/propolis (Hajinezhad et al. 2020), cassava starch/glycerol/beeswax (Auras et al. 2009) and corn starch/beeswax (Ochoa et al. 2017). However, these low EAB data evidence a lack of cohesion between the components of the beeswax, ethanolic propolis extract and cassava starch chains.

**Fig. 2:**
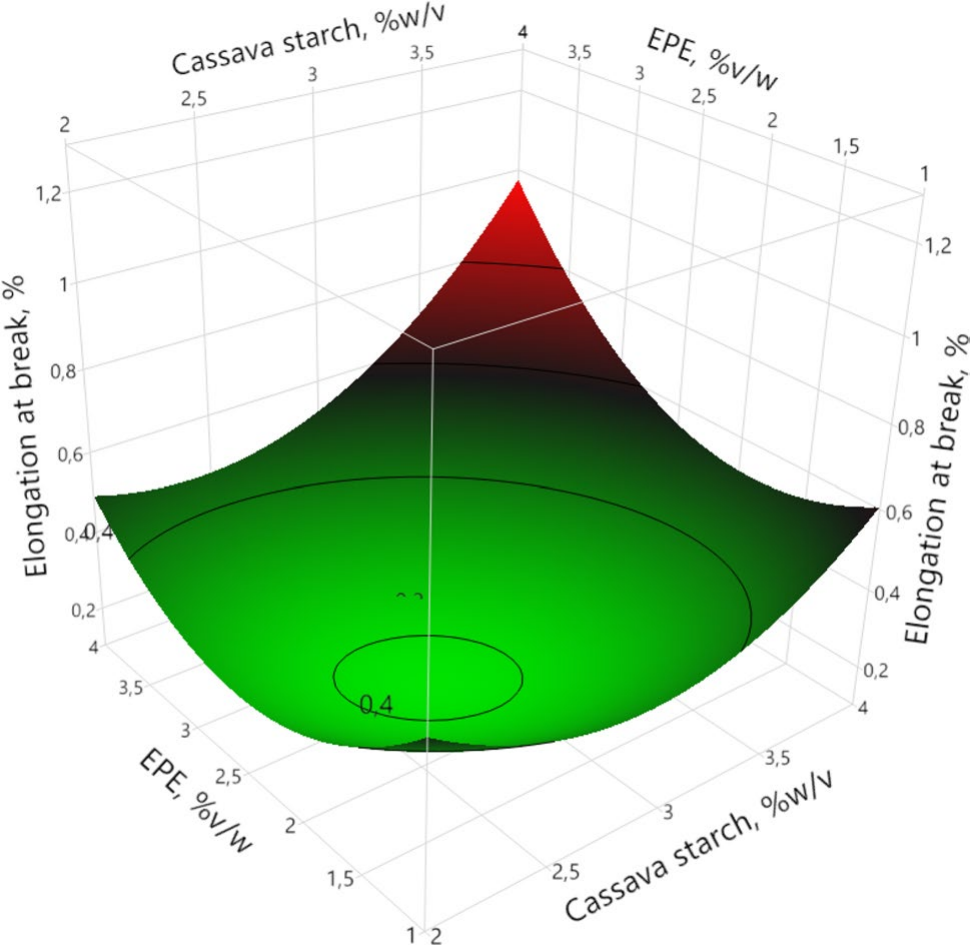
Response surface of elongation at break as a function of native cassava starch and ethanolic propolis extract (EPE)

ANOVA shows that the EAB is influenced significantly (95%) by the lineal factor of cassava starch (*p* = 0.0213), and quadratic factors of cassava starch (*p* = 0.0234) and EPE (*p* = 0.0187). As EPE concentration increases from 1.0 to 2.47%*v/v*, a decrease in EAB was observed (Fig. 2), such a trend has also been reported in films of different matrices with added EPE (Hajinezhad et al. 2020; Mustafa et al. 2020; Pastor et al. 2010), which can be attributed to the weak miscibility of cassava starch and EPE, or producing crystalline zones due to the interactions between EPE components and cassava starch, resulting in less flexibility. As EPE concentration increases from 2.47 to 4.0%*v/w*, an increase in EAB was observed; this trend may be due to the fact that at high concentrations of propolis, it acted as a plasticizing agent, increasing the mobility of the polymer matrix, and resulting in greater flexibility (Ardjoum et al. 2021; Bodini et al. 2013).

#### Young’s modulus (YM)

Young’s modulus (YM) is a fundamental measure of the intrinsic stiffness of films, as the higher YM, the higher the stiffness of the material. YM values were between 3.42 and 22.53 MPa; similar results were reported for films made from cassava starch (Méité et al. 2022), sugar palm (*Arenga pinnata*) starch (Sanyang et al. 2015) and native jicama starch (Wigati et al. 2022).

**Fig. 3:**
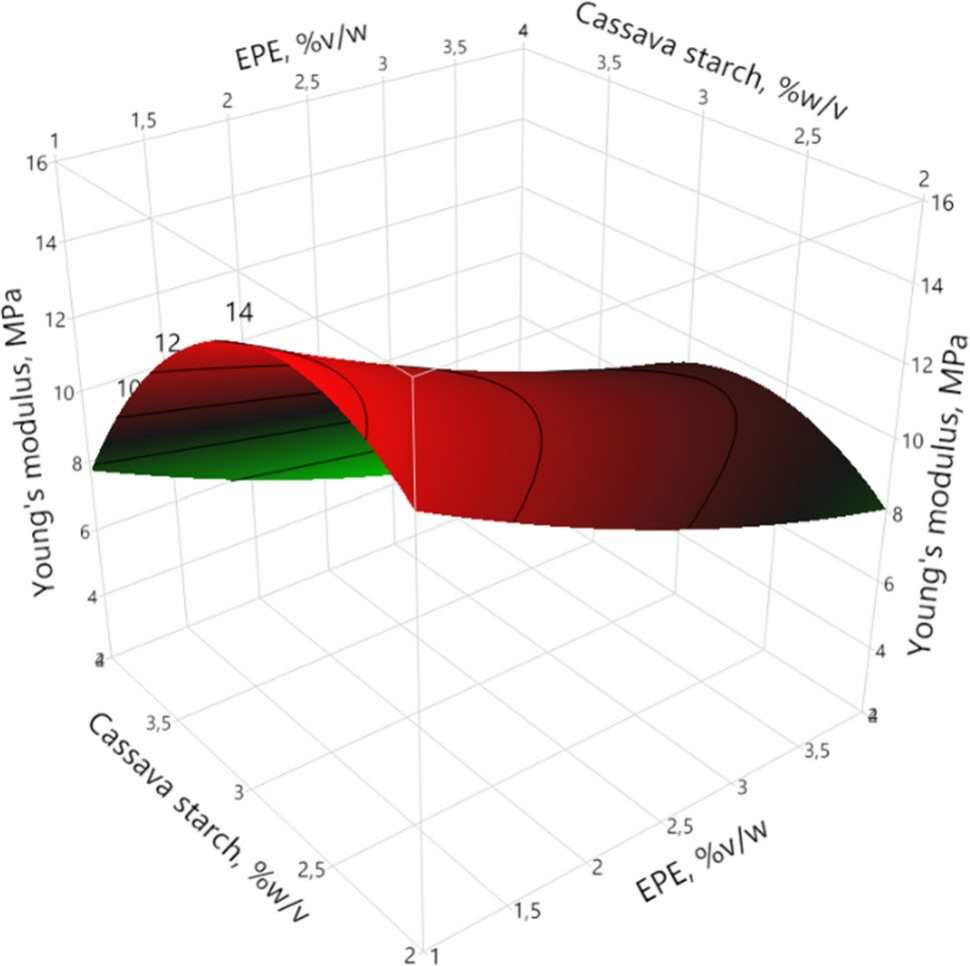
Response surface of Young’s Modulus (YM) as a function of native cassava starch and ethanolic propolis extract (EPE)

ANOVA shows that the YM is influenced significantly (95%) by the lineal factors of cassava starch (*p* = 0.0089) and EPE (*p* = 0.0141), and quadratic factor of cassava starch (*p* = 0.0117). As cassava starch and EPE concentration increases a decrease in YM was observed (Fig. 3), similar results were reported by several authors (De Araújo et al. 2015; Mali et al. 2006). This may be due to the propolis acting as a plasticizing agent, increasing the mobility of the polymer matrix, and resulting in a less rigid film.

### Thermal properties

**Fig. 4:**
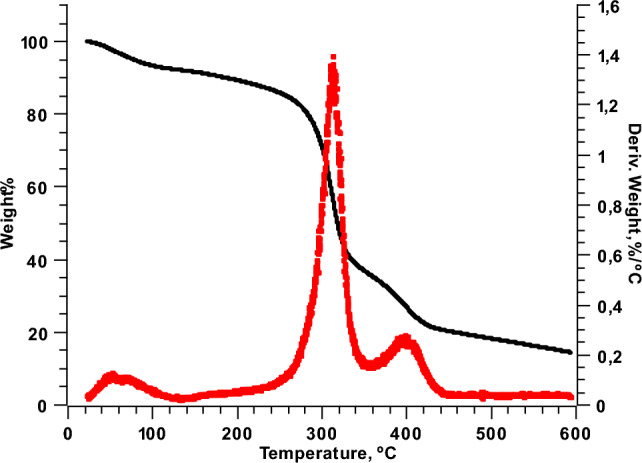
TGA and DTG curves of weight loss rate of cassava starch films incorporated with beeswax and ethanolic propolis extract

All the films showed three main stages of weight loss in TGA/DSC curves in the range from 30 to 440 °C (Fig. 4). First of all, in the temperature range of 30–130 °C, the weight loss of the first stage is mainly due to the evaporation water inter- and intra-molecular hydrogen bonds breaking, and the loss of the volatile compounds of EPE (Ardjoum et al. 2021; Marangoni Júnior et al. 2022). In the second stage, between 200 and 360 °C, the weight loss of the films was related to the decomposition of some low molecular weight polymers in the film matrix, the volatilization of glycerol, decomposition of aromatic rings of phenolic compounds present in the EPE, and the degradation of starch and beeswax (Cheng et al. 2023; Zhang et al. 2018). In this stage, the highest rate of thermal degradation occurred with an approximate temperature of 312 °C and is attributable to the degradation of starch chains. In this phase, ether bonds and unsaturated structures are formed from thermal condensation of the hydroxyl groups of starch chains, thus removing tightly bound water and small molecular size molecules (Ruseckaite and Jiménez 2003). The third stage was found in a range of 360–440 °C, this peak is attributed to the final degradation of the beeswax. According to Reis et al. (2018), the thermal degradation of pure beeswax occurred in three steps, at temperatures of 294, 373 and 440 °C. Furthermore, Cavallaro et al. (2015) reported that the maximum thermal degradation of beeswax was reached at a temperature approximately of 400 °C.

### Anti-fungal activity

The results of the inhibitory effect of edible films based on starch, beeswax and ethanolic propolis extract on *Aspergillus niger* are shown in Table 2. The inhibitory effect of edible films based on starch, beeswax and ethanolic propolis extract was in the range of 12.9–52.4%, with respect to the control. Despite the inhibitory effect achieved, it was not possible to achieve complete inhibition of *Aspergillus niger* fungal growth at the EPE concentrations evaluated. This behavior is similar to that reported in films of different matrices that have used propolis, for example in films based on hydroxypropylmethylcellulose (Pastor et al. 2010), chitosan (De Carli et al. 2022), polylactic acid (Ardjoum et al. 2021), and gelatin (Moreno et al. 2020).

**Table 2:** Inhibitory effect of edible films based on starch, beeswax and ethanolic propolis extract (EPE) against *Aspergillus niger*

Run	Cassava starch, % *w/v*	Beeswax, % *w/w*	EPE, % *v/w*	Inhibition, %
1	3.0	0.9	4.0	49.5 ± 1.0
2	2.0	0.5	2.5	28.9 ± 13.3
3	2.0	0.7	1.0	21.4 ± 4.2
4	2.0	0.7	4.0	32.7 ± 1.3
5	4.0	0.9	2.5	12.9 ± 3.3
6	3.0	0.5	4.0	33.5 ± 8.0
7	4.0	0.5	2.5	39.5 ± 2.8
8	3.0	0.5	1.0	37.5 ± 1.5
9	2.0	0.9	2.5	20.3 ± 8.4
10	3.0	0.9	1.0	19.8 ± 2.7
11	4.0	0.7	1.0	16.0 ± 5.6
12	4.0	0.7	4.0	52.4 ± 4.0
13*	3.0	0.7	2.5	30.4 ± 14.6
14*	3.0	0.7	2.5	25.6 ± 8.2
15*	3.0	0.7	2.5	35.0 ± 10.5

^*^Center points

According to the ANOVA, the percentage inhibition of the films was affected by the linear effect of ethanolic propolis extract (*p* < 0.0007), beeswax (*p* < 0.0300), and the EPE- beeswax interaction effect (*p* < 0.0079). For the interaction effect, it can be explained that at low beeswax level (0.5% *w/w*) the increase of EPE concentration causes a slight increase in the percentage of inhibition (1.67%), while at high beeswax level (0.9% *w/w*) the increase of propolis concentration causes an increase in the inhibition percentage of 222.2% on *Aspergillus niger* fungus. This behavior of the EPE-beeswax interaction effect indicates the synergy that exists between these antimicrobial compounds, especially at high concentrations. The antifungal activity of propolis and its extracts (aqueous or ethanolic) is due to phenolic compounds, specifically the flavonoids and caffeic acid, whose content is related to the phytogeographical origin (Moreno et al. 2020; Pastor et al. 2010). Beeswax contains fatty acids such as palmitic and stearic acids, which have demonstrated antimicrobial properties in some research.

## Conclusion

Suspensions of native cassava starch (2–4%*w/v*), beeswax (0.5–0.9%*w/w*), and ethanolic propolis extract (1–4%*v/w*) form opaque films, with low mechanical properties and highly brittle. According to the behavior of the mechanical properties, the films were not able to form strong molecular bonds between polymer chains, resulting in low resistance. EPE concentration affects the mechanical properties (tensile strength, elongation at break and Young’s modulus) and the cassava starch content affects only the elongation at break and Young's modulus. Regarding thermal properties, all the films showed a similar pattern, presenting three main stages of weight loss, where the greatest weight loss is induced by the cassava starch and beeswax content. On the other hand, films with high beeswax and EPE content inhibit the growth of the *Aspergillus niger* fungus by up to 51%, where the beeswax-EPE interaction has a significant effect. In general, due to the films' mechanical properties and antimicrobial capacity against *Aspergillus niger* fungus, it is recommended that these formulations be used as an edible coating on fruits and vegetables susceptible to fungal growth.

## Data Availability

All data are available in the manuscript.
